# Supplementary material for: The Role of Sphingosine-1-Phosphate Receptor 2 in Mouse Retina Light Responses
Source: Biomolecules. 2023 Nov 23;13(12):1691. doi: 10.3390/biom13121691 (PMC10741782; doi:10.3390/biom13121691)
Supplement: Supplementary file 1 [file biomolecules-13-01691-s001.zip › biomolecules-2665402-supplementary.pdf]

biomolecules

MDPI

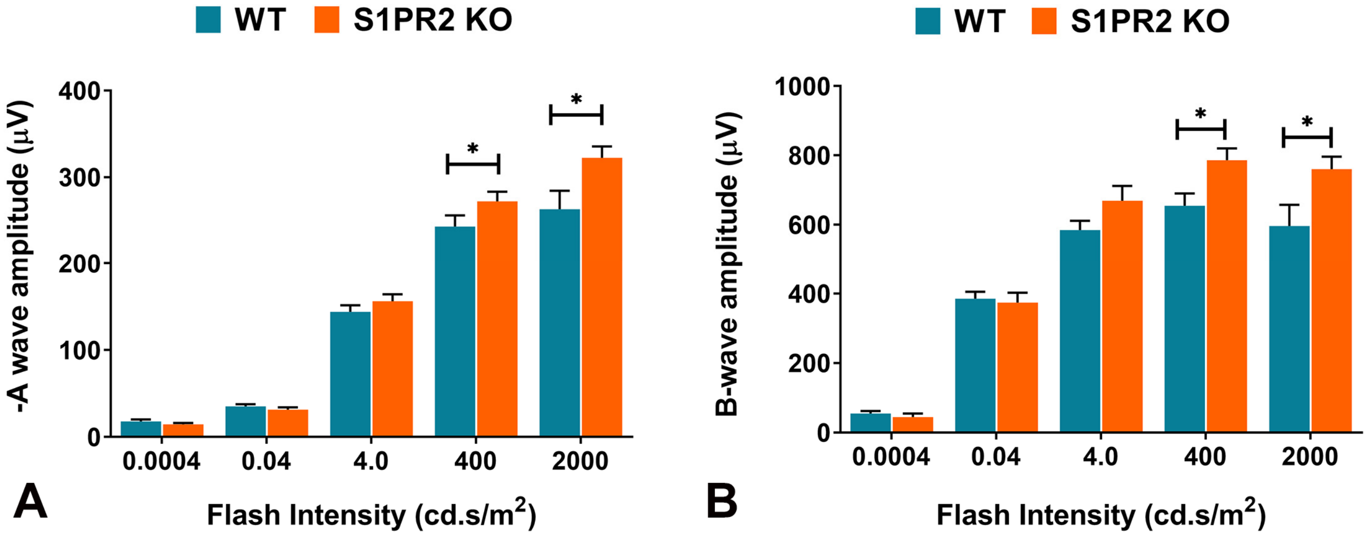


**Supplement Figure S1.** S1PR2 knockout (KO) mice exhibited higher baseline ERG responses than wild-type (WT) at 3 months of age. Shown are statistical analyses of scotopic ERG A- and B-wave amplitudes in 3-month-old WT (blue) and S1PR2 KO (orange) mice. At higher flash intensities (400 and 2000 cd.s/$m^2$), S1PR2 KO mice demonstrated significantly higher mean scotopic A-wave (A) and B-wave (B) ERG responses than WT mice. Shown are mean values ± SEM. *$p < 0.05$ indicate significant differences between groups of WT and S1PR2 KO mice (N = 4 mice per genotype). Abbreviations used: ERG, electroretinography; KO, knockout; WT, wildtype.

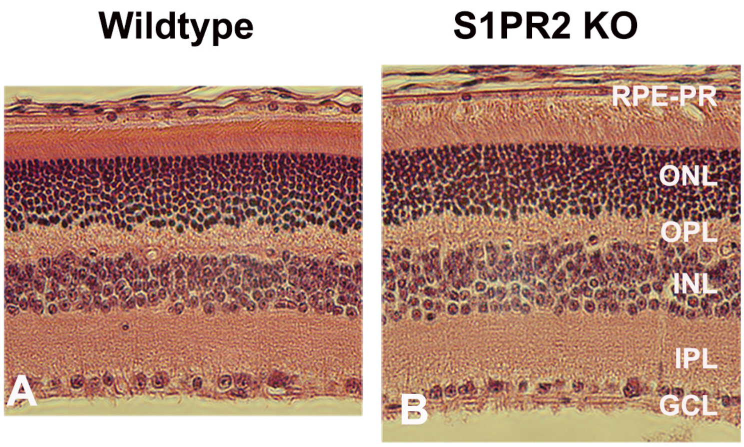


**Supplement Figure S2.** Hematoxylin and Eosin (H&E) stained retinal histological images from the central retina of wildtype (A) and S1P2 KO (B) mice. Images were captured from the same region of the retina and with the same magnification. KO retina appears to be thicker than the wild-type retina. Abbreviations used: RPE-RP, Retina pigment epithelium and Photoreceptor outer segments; ONL, outer nuclear layer; OPL, outer plexiform layer; INL, inner nuclear layer; IPL, inner plexiform layer; GCL, ganglion cell layer.

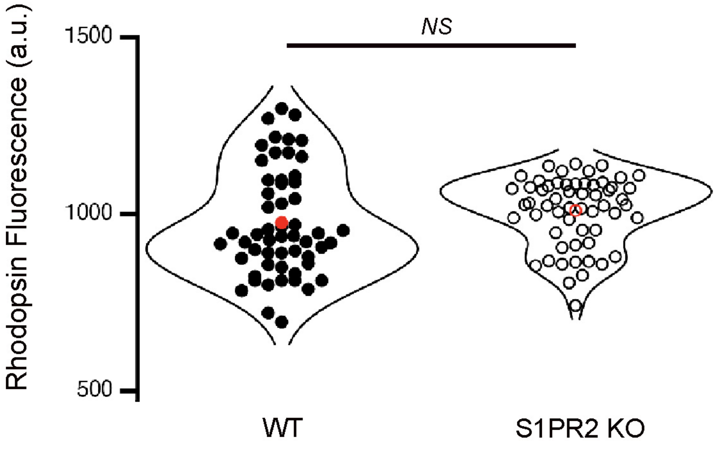


**Supplement Figure S3.** The retinas from S1PR2 KO mice retained WT rhodopsin expression. Quantitative analyses of IHC for rhodopsin as a measure of fluorescent intensity that contained rods. Images were captured from the same region of the retina and with the same magnification. Abbreviations used: KO, knockout; NS, not significantly different; WT, wildtype. Data represents the average area of expression in 4–6 animals sampled per condition in 3–5 independent experiments.

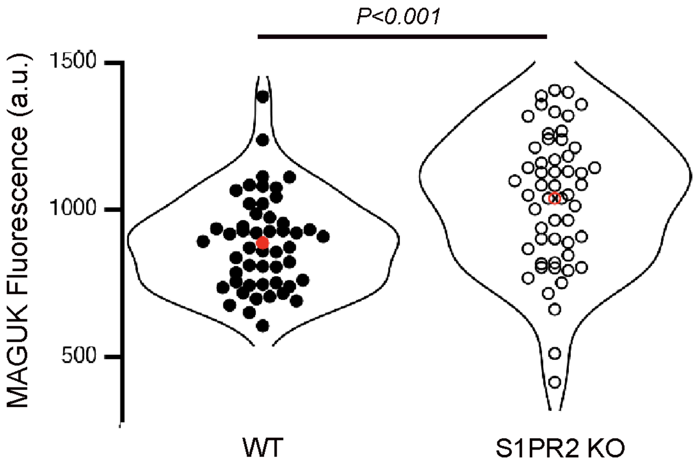


**Supplement Figure S4.** Immunostaining demonstrates expression of MAGUK in dendrites of bipolar cells of WT and S1PR2 KO mice. Images were captured from the same region of the retina and with the same magnification. The fluorescent intensity of MAGUK in the KO retina appears to be significantly higher than in the wild-type retina. Abbreviations used: KO, knockout; NS, not significantly different; WT, wildtype. Data represents the average area of expression in 4–6 animals sampled per condition in 3–5 independent experiments. Statistical relevance was determined using a two-tailed *t*-test; $p < 0.001$.

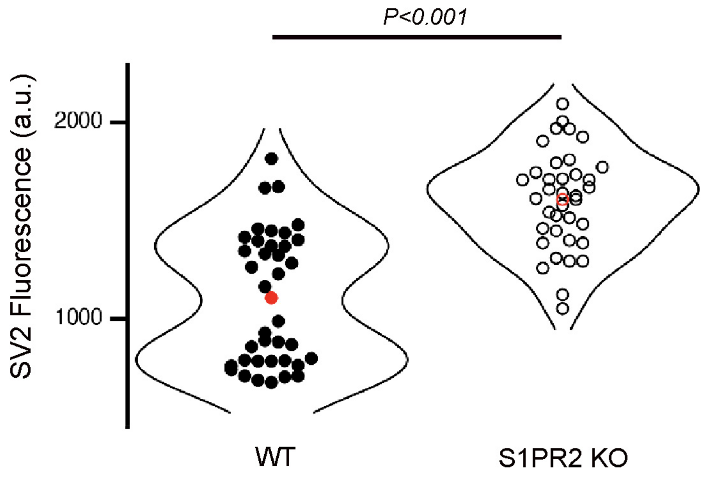


**Supplement Figure S5.** Immunostaining demonstrates the expression of SV2 in the synaptic terminal of photoreceptors of WT and S1PR2 KO mice. Images were captured from the same region of the retina and with the same magnification. The fluorescent intensity of SV2 in the KO retina appears to be significantly higher than in the wild-type retina. Abbreviations used: KO, knockout; NS, not significantly different; WT, wildtype. Data represent the average area of expression in 6–9 animals sampled per condition in 3–5 independent experiments. Statistical relevance was determined using a two-tailed *t*-test; $p < 0.001$.

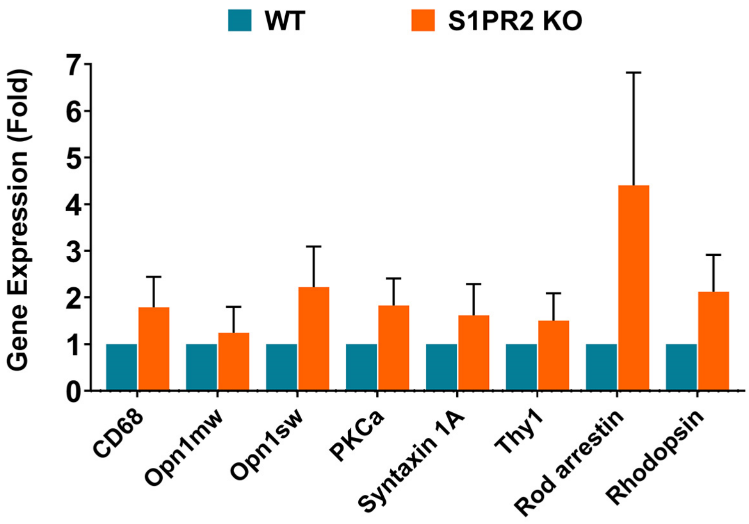


**Supplement Figure S6.** Expression of retinal markers in WT and S1PR2 KO mice. Statistical analysis of gene expression of retinal markers in the retinas of WT (blue) and S1PR2 KO (orange) mice by RT-qPCR. Retinal expression of CD68/LAMP4, Opn1mw, Opn1sw, PKC$\alpha$, syntaxin 1A, Thy1/CD90, rod arrestin, and rhodopsin did not significantly differ between WT and S1PR2 KO mice. Shown is the mean fold value over control ± SEM (N= 6). Abbreviations used: CD68/LAMP4, cluster of differentiation 68/lysosomal-associated membrane protein 4; KO, knockout; Opn1mw, medium wave-sensitive opsin 1; Opn1sw, short wave-sensitive opsin 1; PKC$\alpha$, protein kinase C alpha; Trpm1, Thy1/CD90, Thy1 cell surface antigen/cluster of differentiation 90; WT, wildtype.
